# On Certain Forms of Headache

**Published:** 1848-06

**Authors:** 


*On Certain Forms of Headache.*—Dr. P. Murphy in a paper on this subject read before the South London Medical Society, (Nov. 25th, 1847,) after alluding to the frequent occurrence of headachh, stated that his intention was to confine himself to the consideration of those varieties originating from disease of bone, or its periosteal covering; from disease of fibrous structures, or rheumatism; from affections of the occipital nerves, or from deficiency or excess of blood within the cranium.

Periostitis of the cranium is scarcely met with unless after a mercurial course, the constitution being almost always scrofulous, and is generally found to invade either the coronal or parietal bones, which the author attributed to their less protected situation. The diagnosis of this form of headache, although not difficult, has been at times erroneous. The pain is severe, and confined to one or more spaces of the above-mentioned bones: is increased by warmth at night, by stimulating drinks, by exposure to cold and pressure; and a raised surface can be detected by the finger at the spot in pain: and, on inquiry, it will be found that more than one course of mercury has been undergone. It occurs chiefly in men below forty years of age, and in the degraded class of females. The treatment should consist in the application of the Empl. c. Hydr. spread on thick leather, to the part, which both protects it from cold, and removes the effusion of lymph. The exhibition of the iodide of potassium, combined with morphia and tinct. digitalis, is highly useful.

Another form of head-ache, the rheumatic or fibrous, not very common, is located in the tendon of the occipito-frontalis, the temporal aponeurosis, and tendinous insertions of muscles at the back of the head: it is usually preceded by rheumatism of other parts, and is increased by muscular movements. Warm coverings, when they can be applied, usually relieve it; also sinapisms; and, if the pain is very severe, leeches and cupping, and a few doses of calomel with opium, seldom fail to give relief. Gout may also attack the same parts, but be diagnosed by our previous acquaintance with the habits of the patient. In both cases the pain is intermitting, changes its locality, is felt to be external, and the health is not affected.

The next form belongs to the class of spinal irritation, is very frequent, and met with exclusively in females during the menstrual periods, and attacks mostly the left side of the head; the pain is intermittent, shooting, and lancinating; may be fixed for days, and is most severe at the temple (when it is termed clavus hystericus), and next at the parietal protuberance and occiput: it proceeds from the sub-occipital nerve, and, if the exit of the nerve is pressed upon, pain more or less severe is complained of, extending along the whole course or at certain sites only of the nerve—as at the temple, nape of the neck, parietal protuberance, &c.: it is usually increased during the menstrual period, and is generally a complaint of unmarried females between the twenty-third aud thirty-fifth years of life, and is indubitably a form of hysteria. The menses are usually profuse or difficult, the bladder irritable, and there are ill-defined painful sensations about the pelvis; other forms of nueralgia often co-exist. The irritation of the sub-occipital nerve must be traced to the ovaries, being only present where these exist, and while capable of fulfilling the functions of menstruation; and our treatment must be primarily directed, to remove any congestion or irritation of these peculiar organs, and secondarily to lessen the pain of the nerves. The author advises the daily use of hip-baths or sea-bathing where possible; attention to prevent accumulations in the rectum; abstinence from stimulants; mental employment; inf. valerian c. digitalis, with pills of assafœtida; occasionally general or only local bleeding; and, when these fail, a gentle mercurial action, the cold bath being during the time omitted. As local means he recommends belladonna plasters, veratrine ointment, sinapisms, or blistering. Where, however, the patient is exhausted by leucorrhœa or profuse menstruation, with symptoms of chronic inflammation of the womb or ovaries, the treatment becomes more doubtful; but the author prefers the trial of a tonic treatment, and advises the exhibition of the valerianate of zinc and quinine, as especially efficacious, and the sulphate of iron in infusion of valerian when there are evidences of confirmed chlorosis. This head-ache may be termed the *nervous* head-ache: it also assumes another form, which may be termed the *cutaneous* head-ache, and is the hemicrania of our forefathers: it seems to be located in the integuments of one half—usually the left side—of the head which is so exquisitely sensible as scarcely to bear the least touch of the finger, and the pain never passes the mesial line.

Another form of head-ache is that arising from deficiency of blood within the cranium, and coming on after hemorrhages, exhausting discharges, or any other debilitating causes: the best examples occur in chlorosis. It is increased by the erect, diminished by the recumbent posture; is not a very painful form, but is often attended with impaired vision: its cause may be traced to diminished muscular power of the heart, which palpitates on slight exertion: there is also dyspnœa, pale face, and other symptoms of a feeble circulation, with a sinking pain at the epigastrium, and craving appetite. If the true cause of this head-ache is mistaken, and depletion used, paralysis has been known to supervene, but if the debility be removed, the muscular power of the heart is easily increased; and the most useful remedies are, steel by itself or combined with quina, full diet, and the recumbent posture.

The last form of head-ache alluded to by the author arises from excess of blood, and may exist as a passive or congested, or as an active or inflammatory state. The former, arising from the various known causes of congestion, is diagnosed by the constant heavy pain at the anterior part of the head, increased by the recumbent posture, sense of chilliness, slow feeble pulse, tendency to vomiting, and pain in the lumbar region, caused by congestion of the spinal cord. It is a dangerous form of headache, and has in the depressing diseases proved in a few hours fatal, but in other cases has lasted weeks without much mischief. The treatment should be to induce reaction as soon as possible by the warm-bath or an emetic. The author deprecated the usual attempts to arrest vomiting during the invasion of fevers. If the headache persists with hot skin, leeches to the inner nares will be found of value; applied to the temple, they debilitate without relieving this pain of head, and they are altogether inadmissible when this co-exists with typhus or scarlatina. Blisters may also be applied, and diaphoresis produced by the usual means; cold applications to the head, the author considered useless, and even likely to increase congestion. Care was also requisite that mere congestion should not by the use of stimuli be forced into inflammation, which is the next stage if resolution or fatal termination does not take place. The author regarded idiopathic phrenitis as a most rare disease, and hydrocephalus acutus as congestion and not inflammation. Phrenitis was well marked by the tensive pain increased on stooping, by the bright eye, hot skin, nausea, and vomiting, tendency to delirium, and occasional twitching of the muscles of the face; the most active antiphlogistic measures should be used.

There were other forms of headache easy of diagnosis, but of these the author would only mention the constant pain of head in children, with emaciation and want of sleep, and which diagnosed tubercles of the brain. In the headache of pregnant females referred to the centre of the head, and attended with a remarkably slow pulse, and in which if bleeding is neglected, convulsions, abortion, and too often death, are apt to supervene; and lastly the pain of the head occurring after a nights debauch, the cause of which, whether in the stomach or affected organ, the author considered not to have been sufficiently investigated.

The President inquired of the author his means of judging in cases of headache whether the treatment should be depletory or stimulating, as in his own practice he had at times found it extremely difficult to decide, and was often obliged to try, as it were, a tentative treatment.

Dr. P. Murphy thought we should not often be led astray if attention was paid to certain points. If a patient complained of the pain of the head coming on and increasing after getting up in the moring, or assuming the erect position, and relieved on resuming the horizontal; if troubled with dimness of sight, faintness, perspiration, palpitations on exertion, cramps of legs, depression of spirits, frightful dreams, &c., he concluded a tonic treatment would be useful; headache from congestion is, moreover, a rare disease. In answer to Mr. Wright, his usual practice in clavus hystericus was to commence with tonics, which usually affected a cure. Still in some cases he resorted to cupping at the back of the neck with the greatest benefit, after the former plan had failed. In cases of young females where signs of congestion about the womb existed, leeches to the groins were often useful.

Dr. Sylvester agreed in the value of Dr. Murphy's Diagnostic symptoms, and alluded to the existence of the venous bruit, which, when present, always indicated an anæmial condition of system, and contraindicated depletion, but is benefitted by a tonic plan; as a general rule, he thought it wiser not to deplete in headache. He did not agree with the author that the pain of syphilitic headache occurred generally at the front or most exposed portions of bone; he thought it was more general about the muscular insertions at the back of the neck, and in parts well covered with muscles; there appeared to be an electric attraction in the poison for certain parts of bones.—*London Med. Gaz.—Am. Jour. of Med. Science.*

